# Comparative genomic insights into carbapenem-resistant versus susceptible *Acinetobacter baumannii* in Shanghai hospital settings: a multi-center molecular surveillance study

**DOI:** 10.3389/fcimb.2026.1878371

**Published:** 2026-07-31

**Authors:** Yue Zhang, Yanru Liang, Yuanping Wang, Yiying Xu, Ye Qiu, Bing Zhao, Yifeng Shen, Xiao Wang

**Affiliations:** 1 Shanghai Pudong New Area Center for Disease Control and Prevention (Shanghai Pudong New Area Health Supervision Institute), Shanghai, China; 2 Institute of Respiratory Medicine, Chinese Academy of Medical Sciences, Peking Union Medical College, Beijing, China

**Keywords:** antimicrobial resistance, Carbapenem-resistant *Acinetobacter baumannii*, medical institution environment, phylogeny, ST164

## Abstract

**Background:**

Carbapenem-resistant *Acinetobacter baumannii* (CRAB) is priority pathogen for nosocomial infection control worldwide. This study aimed to elucidate differences in distribution, genotyping and antimicrobial resistance patterns between CRAB and carbapenem-susceptible *Acinetobacter baumannii* (CSAB) strains isolated from healthcare environments.

**Materials and methods:**

A total of 1,812 environmental samples were collected from 10 medical institutions in Pudong New Area between June and December 2024 for the identification of *A. baumannii*. Antimicrobial susceptibility testing, whole-genome sequencing, and bioinformatic analysis were performed to reveal molecular characteristics of *A. baumannii.*

**Results:**

A total of 81 *A. baumannii* strains were detected, with 39.51% CRAB (*n* = 32) and 60.49% CSAB (*n* = 49). CRAB strains were highly prevalent in intensive care units (ICUs, accounting for 83.33%) and patient-contact items, whereas CSAB were more evenly distributed. MLST analysis identified ST2 as dominant CRAB clone (78.13%), followed by ST164 (18.75%). CRAB isolates demonstrated 100% resistance to multiple antibiotics such as piperacillin/tazobactam and ciprofloxacin, whereas all CSAB isolates were susceptible to tested antibiotics except one ampicillin/sulbactam-resistant isolate. Carbapenem resistance in CRAB was predominantly mediated by class D carbapenemases, with carriage rate of the *bla*_OXA-23_ gene reaching 96.88%. Notably, six ST164 isolates co-harbored class B and class D carbapenemase genes, which may elevate resistance severity. Bioinformatic analysis indicated that 22.20% of resistance plasmids in CRAB were predicted to be conjugative. CRAB exhibited a more diverse and extensive virulence gene profile than CSAB strains.

**Conclusions:**

The ICU environment was critical for CRAB control. ST2 and ST164 represented clones worthy of attention. CRAB exhibited stronger resistance, harbored more putatively conjugative plasmids and richer virulence-associated genes than CSAB, highlighting the need for targeted disinfection and surveillance of prevalent clones.

## 1 Introduction

*Acinetobacter baumannii* is an important nosocomial pathogen that can cause severe infections such as pneumonia, bloodstream infections, meningitis, and skin infections in individuals (Müller et al., 2023; Tamma et al., 2024). *A. baumannii* exhibited extremely strong viability-it could tolerate a broad range of temperatures and humidity, and survive on dry skin or object surfaces for months. This characteristic facilitates its transmission in healthcare environments, leading to infection epidemics and endemic persistence (Hassan et al., 2021; Müller et al., 2023). The increasingly severe drug resistance situation is another important reason for the continuous prevalence of *A. baumannii*. Reports indicate that *A. baumannii* is becoming more resistant to multiple antibiotics. With its high detection rate, strong adaptability, and severe drug resistance, *A. baumannii* has been listed as a priority drug-resistant pathogen by the World Health Organization (WHO), posing an urgent public health problem globally (Chen et al., 2025; Sati et al., 2025).

Carbapenems are key antibiotics for the clinical treatment of *A. baumannii* infections. However, with the irrational use of antibiotics, Carbapenem-resistant *Acinetobacter baumannii* (CRAB) has been constantly emerging. Studies have shown that the Centers for Disease Control and Prevention (CDC) of the United States has long classified CRAB as an “urgent threat,” the highest level in its risk classification (Watkins and Bonomo, 2023; Kubin et al., 2025). Among *A. baumannii* strains collected from three major hospitals in Jordan, 90.6% were resistant to carbapenems (Ababneh et al., 2021). The epidemic trend of CRAB in China is equally worrying. Among 77 intensive care unit (ICU) wards of hospitals in different provinces surveyed, 71.4% had CRAB prevalence, and clonal transmission of CRAB was found in 37.6% of ICUs. The resistance of these CRAB strains is mainly related to the production of carbapenemases, especially the class D β-lactamase encoded by *bla*_OXA-23_. *bla*_OXA-23_ can be vertically or horizontally transmitted among strains through chromosomes or plasmids, which is the reason for its wide distribution and transmission. It is worth noting that CRAB is often multidrug-resistant and carries more resistance genes and virulence genes compared with carbapenem-susceptible *Acinetobacter baumannii* (CSAB) (Chen et al., 2025). Clinical data also confirm that compared with CSAB infections, patients infected with CRAB have a significantly heavier disease burden and a higher risk of death (Pogue et al., 2022).

As a key site for the transmission and colonization of *A. baumannii*, medical institution environments-especially object surfaces such as medical devices and doctor-patient direct contact items-are important reservoirs of this bacterium. Studies have confirmed that *A. baumannii* in the environment can achieve nosocomial transmission through poor hand hygiene of medical staff and cross-contact of contaminated items, which is a core risk factor for nosocomial clustered infections or even outbreaks. At present, most studies on *A. baumannii* center on clinically isolated strains, and attention to strains in healthcare environments is gradually increasing. However, existing studies mostly focused on the single detection of CRAB and screening of resistance genes (Wong et al., 2022), lacking systematic comparative analysis of CRAB and CSAB in healthcare environments. Clarifying the distribution differences between CRAB and CSAB, and understanding the similarities and differences in drug resistance and epidemic sequence type (ST) between the two strains, can provide key evidence for tracing infection sources and determining transmission chains, thereby optimizing healthcare environment disinfection strategies and infection control measures. Based on this, this study selected 10 medical institutions in Pudong New Area as research objects, and systematically collected surface samples from different departments. The drug resistance and whole-genome sequence characteristics of isolated *A. baumannii* were analyzed, and the distribution characteristics, antimicrobial resistance, ST profiles, and plasmid carriage of CRAB and CSAB were compared, aiming to provide scientific basis for the environmental prevention and control of CRAB in regional medical institutions and help reduce the risk of nosocomial infections caused by *A. baumannii*.

## 2 Materials and methods

### 2.1 Sample source

According to the drug-resistant bacteria risk monitoring plan for medical institutions in Pudong New Area, all environmental samples were collected from different departments of 10 medical institutions in different geographical locations. The sampling frequency was one collection per quarter for each hospital. 1,812 environmental samples were obtained as test objects between June and December 2024. Detailed sampling quantities and departmental distributions were summarized in **Supplementary Table 1**. The specific on-site collection method was in accordance with GB 15982-2012 “Hygienic Standard for Hospital Disinfection.”

### 2.2 Identification of *Acinetobacter baumannii*

Samples were inoculated into double-strength TSB enrichment broth and cultured at 36 ± 1°C for 48h. The enrichment broth was inoculated on Acinetobacter chromogenic plates and blood plates, cultured at 36 ± 1°C for 24-48h, and suspected colonies were selected for Gram staining, oxidase, and other biochemical tests. Meanwhile, secondary confirmation was performed by mass spectrometry identification-colonies with a score >2.1 were identified as target strains. Pure colonies were preserved in 3% glycerol brain-heart infusion broth at -80°C for later use.

### 2.3 Antimicrobial susceptibility testing

AST Panel For Aerobic Gram Negative bacilli (Fosun Diagnostics Technology Co, Shanghai, China) was used to determine the resistance profile of *A. baumannii*. Antimicrobial susceptibility testing detected minimum inhibitory concentrations (MICs) values of 16 types of antibiotics, including Piperacillin/Tazobactam, Ampicillin/Sulbactam, Cefoperazone/Sulbactam, Cefotaxime, Ceftazidime, Cefepime, Imipenem, Meropenem, Minocycline, Tigecycline, Gentamicin, Amikacin, Trimethoprim/, Sulfamethoxazole, Polymyxin B, Ciprofloxacin and Levofloxacin. The *E. coli* ATCC^®^25922TM strain was employed as the quality control strain. Results were categorized as susceptible (S), intermediate (I), or resistant (R) based on the Clinical and Laboratory Standards Institute (CLSI) standards.

### 2.4 Whole genome sequencing

*A. baumannii* strains were retrieved and purified by streaking on blood agar plates. Genomic nucleic was extracted using BG-Nege-96 fully automatic nucleic acid extraction instrument and its corresponding proprietary reagents (Shanghai Bojie Medical Technology Co., Ltd, China).The sequence was performed on the DNA library generated by Whole-genome DNA Library Construction Kit (Shanghai Bojie Medical Technology Co., Ltd, China) using MGISEQ-T7 platform (MGI Tech Co., Ltd, China). Raw data from each sample were filtered using Fastp software (version 0.23.2) to trim adapter sequences and discard low-quality reads. SPAdes software (version v3.15.5) was performed for reference-free assembly of bacterial samples. Kraken (version 2.1.2) was employed to annotate the assembled data and non-target bacterial sequences were filtered out. All sequencing data have been uploaded to the NCBI database under BioProject ID: PRJNA1390075.

### 2.5 Bioinformatics analysis

Multilocus Sequence Typing (MLST) of *A. baumannii* were obtained based on the PubMLST platform (2025, https://pubmlst.org/). The 7 housekeeping genes used for MLST analysis based on the Pasteur scheme were *cpn60*, *fusA*, *gltA*, *pyrG*, *recA*, *rplB* and *rpoB*, while those based on the Oxford scheme were *gltA*, *gyrB*, *gdhB*, *recA*, *cpn60*, *gpi* and *rpoD*. Based on MLST, Grapetree was used to generate and display minimum-spanning trees (MST) of study data. The ABRIcate (v1.0.1) tool (https://github.com/tseemann/abricate) was used to identify resistance genes and virulence genes of strains based on the ResFinder database and VFDB database, respectively. Core-genome alignment was executed with Snippy (v4.6.0), using *A. baumannii* ATCC 19606 (GenBank accession No. GCF_009035845.1) as the reference genome. Snp-dists (v0.8.2) was utilized to calculate pairwise allelic single-nucleotide polymorphism (SNP) distances from this matrix. A maximum-likelihood phylogenetic tree was reconstructed based on core-genome single-nucleotide polymorphisms (core-SNPs), which was visualized through the Chiplot online platform (https://www.chiplot.online/tvbot.html) (Xie et al., 2023). MOB-Suite (v3.19) software was used to identify and reconstruct plasmid sequences from genome assembly data, and the conjugative transfer ability of plasmids was predicted via the MOB-typer module. All analytical parameters were set to default values.

### 2.6 Statistical analysis

Comprehensive statistical analyses were performed with the aid of SPSS statistical software (Version 22) and GraphPad Prism (Version 11.0). For the analysis of categorical variables, either Pearson’s chi-square test or Fisher’s exact test was selected based on the sample size and expected cell frequencies. The consistency between resistance phenotypes and resistance genes was evaluated using Cohen’s kappa statistics. The Kolmogorov-Smirnov test was implemented to evaluate whether the continuous data followed a normal distribution. In the case of two-group comparisons, the parametric unpaired t-test was applied for normally distributed data, whereas the nonparametric Mann-Whitney U test was used for data that deviated from normality. Two-sided and *P* < 0.05 was considered to be statistically significant.

## 3 Results

### 3.1 Distribution of *Acinetobacter baumannii*

**Figure 1:**
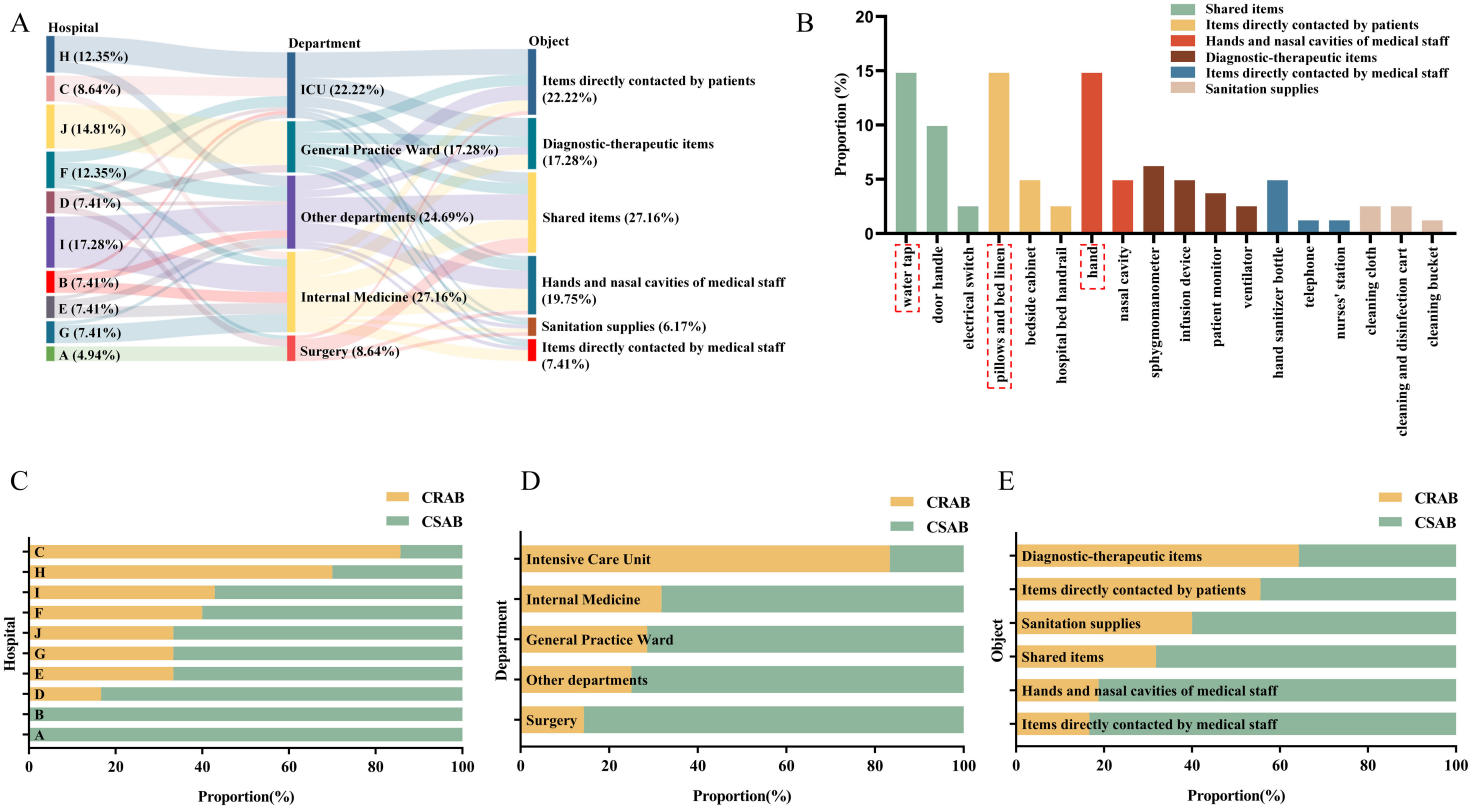
Distribution of *A. baumannii* isolates in the medical institutions environment. **(A)** The Sankey diagram depicts the distribution profile of all 81 A*. baumannii s*trains across 10 medical institutions, 5 departments, and 6 Objects. The thickness of each line is proportional to the quantity **(B)** Distribution of 81 A*. baumannii* strains in specific sampling sites (The top three sites are marked with red dashed boxes); **(C)** Comparison of the proportions of CRAB and CSAB among different medical institutions; **(D)** Comparison of the proportions of CRAB and CSAB among different departments; **(E)** Comparison of the proportions of CRAB and CSAB among different objects.

The overall detection rate of *A. baumannii* was 4.47% (81/1812), and the recovered isolates displayed distinct distribution across different hospitals. Hospital I had the highest proportion at 17.28%, whereas Hospital A presented the lowest proportion of only 4.94%. By department, the proportion of *A. baumannii* isolates was the highest in Internal Medicine (27.16%), compared with merely 8.64% in Surgery. Spatial distribution analysis demonstrated that *A. baumannii* was widely present in shared items, items directly contacted by patients, and hands/nasal cavities of medical staff (**Figure 1A**). Among them, water taps, pillows and bed linen, and medical staff’s hands were hotspots of *A. baumannii* contamination (**Figure 1B**).

Among the 81 A*. baumannii* strains, CRAB accounted for 39.51% (*n* = 32) and CSAB accounted for 60.49% (*n* = 49). The two also showed marked disparities in their distribution across medical institutes, departments, and objects (**Table 1**). With regard to institutional distribution, Hospital H recorded the highest proportion of CRAB isolates at 21.88%, whereas Hospitals I and J shared the highest proportion of CSAB isolates at 16.33% each. Among *A. baumannii* strains in Hospital C, CRAB isolates accounted for more than 85%, while no CRAB was detected in Hospitals A and B (**Figure 1C**). For departmental distribution, CRAB isolates were predominantly present in ICUs (46.88%), while CSAB isolates were relatively evenly distributed in other departments such as Internal Medicine. A key finding was that among all *A. baumannii* strains in ICU environments, CRAB accounted for as high as 83.33%, which was significantly higher than CSAB (16.67%). In other departments such as Surgery, CSAB was more numerous than CRAB (**Figure 1D**). In terms of objects, CRAB isolates were concentrated in items directly contacted by patients, while CSAB was primarily found on shared items, etc. The proportion of CRAB isolates in diagnostic-therapeutic items and items directly contacted by patients was significantly higher than that of CSAB. Most *A. baumannii* strains detected in hands/nasal cavities of medical staff and items directly contacted by medical staff were CSAB (**Figure 1E**).

**Table 1:** Multidimensional distribution of CRAB and CSAB in the medical institutions environment.

Items		Number (%)		X^2^	*P* value
		CRAB (*n* = 32)	CSAB (*n* = 49)		
Hospital	A	0 (0.00)	4 (8.16)	18.435	0.030*
	B	0 (0.00)	6 (12.24)		
	C	6 (18.75)	1 (2.04)		
	D	1 (3.13)	5 (10.20)		
	E	2 (6.25)	4 (8.16)		
	F	4 (12.50)	6 (12.24)		
	G	2 (6.25)	4 (8.16)		
	H	7 (21.88)	3 (6.12)		
	I	6 (18.75)	8 (16.33)		
	J	4 (12.50)	8 (16.33)		
Department	ICU	15 (46.88)	3 (6.12)	19.336	0.001*
	Internal Medicine	7 (21.88)	15 (30.61)		
	Surgery	1 (3.13)	6 (12.24)		
	General Practice Ward	4 (12.50)	10 (20.41)		
	Other departments	5 (15.63)	15 (30.61)		
Object	Items directly contacted by patients	10 (31.25)	8 (16.33)	10.276	0.068
	Diagnostic-therapeutic items	9 (28.13)	5 (10.20)		
	Shared items	7 (21.88)	15 (30.61)		
	Hands and nasal cavities of medical staff	3 (9.38)	13 (26.53)		
	Sanitation supplies	2 (6.25)	3 (6.12)		
	Items directly contacted by medical staff	1 (3.13)	5 (10.20)		

*Bold value, *P* < 0.05. ICU, intensive care unit.

### 3.2 MLST typing of *Acinetobacter baumannii*

**Figure 2:**
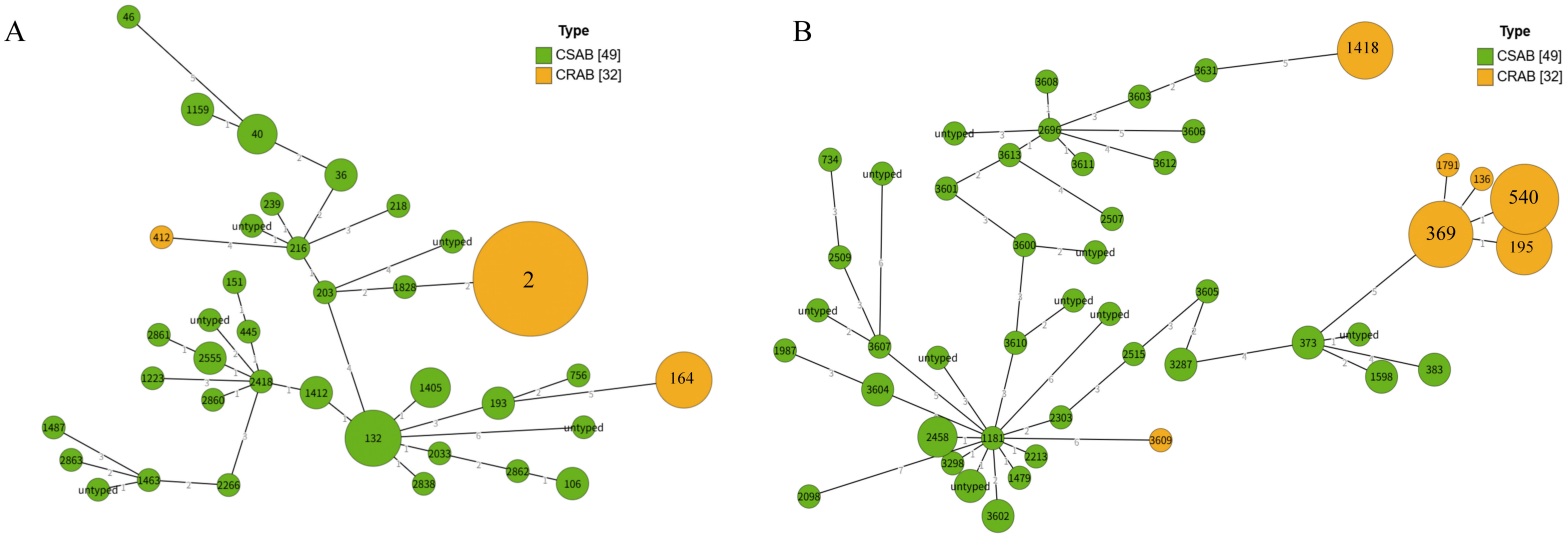
MLST typing of *A. baumannii* isolates. **(A)** Minimum Spanning Tree of CRAB and CSAB based on Pasteur MLST Scheme; MLST types are labeled inside circles; circle size corresponds to the number of isolates for each MLST type, with larger circles indicating higher counts. **(B)** Minimum Spanning Tree of CRAB and CSAB based on Oxford MLST Scheme; MLST types are labeled inside circles; circle size corresponds to the number of isolates for each MLST type, with larger circles indicating higher counts.

The Pasteur and Oxford schemes were used to analyze the MLST of the whole-genome sequences of 81 *A. baumannii* strains. Typing results based on the Pasteur scheme showed that the 81 *A. baumannii* strains were assigned to 32 distinct sequence types (STs). The 32 CRAB strains fell into only 3 types, with the ST2 clone accounting for the highest proportion (78.13%), consistent with the dominant clone type worldwide. Six strains (18.75%) belonged to ST164, and only 1 strain was classified as ST412. Compared with CRAB, CSAB exhibited a substantially broader ST distribution, encompassing a total of 29 distinct STs. ST132 was the most prevalent type, accounting for 12.24% of all CSAB strains (**Figure 2A**).

Based on the Oxford scheme, typing results showed that the 81 *A. baumannii* strains belonged to 37 STs. There were no overlapping types between CRAB and CSAB. CRAB isolates were restricted to 7 of these types, with ST540 and ST369 as the dominant types, accounting for 28.13% and 25.00% respectively. Nodes such as ST540, ST369, ST195, ST1791, and ST136 on the right were closely connected. ST1418 and ST3609 were positioned on distinct distantly related clades. Similar to the Pasteur typing results, CSAB covered multiple genetic branches of the MST with scattered STs, indicating high genetic diversity (**Figure 2B**).

### 3.3 Clustering analysis of *Acinetobacter baumannii*

**Figure 3:**
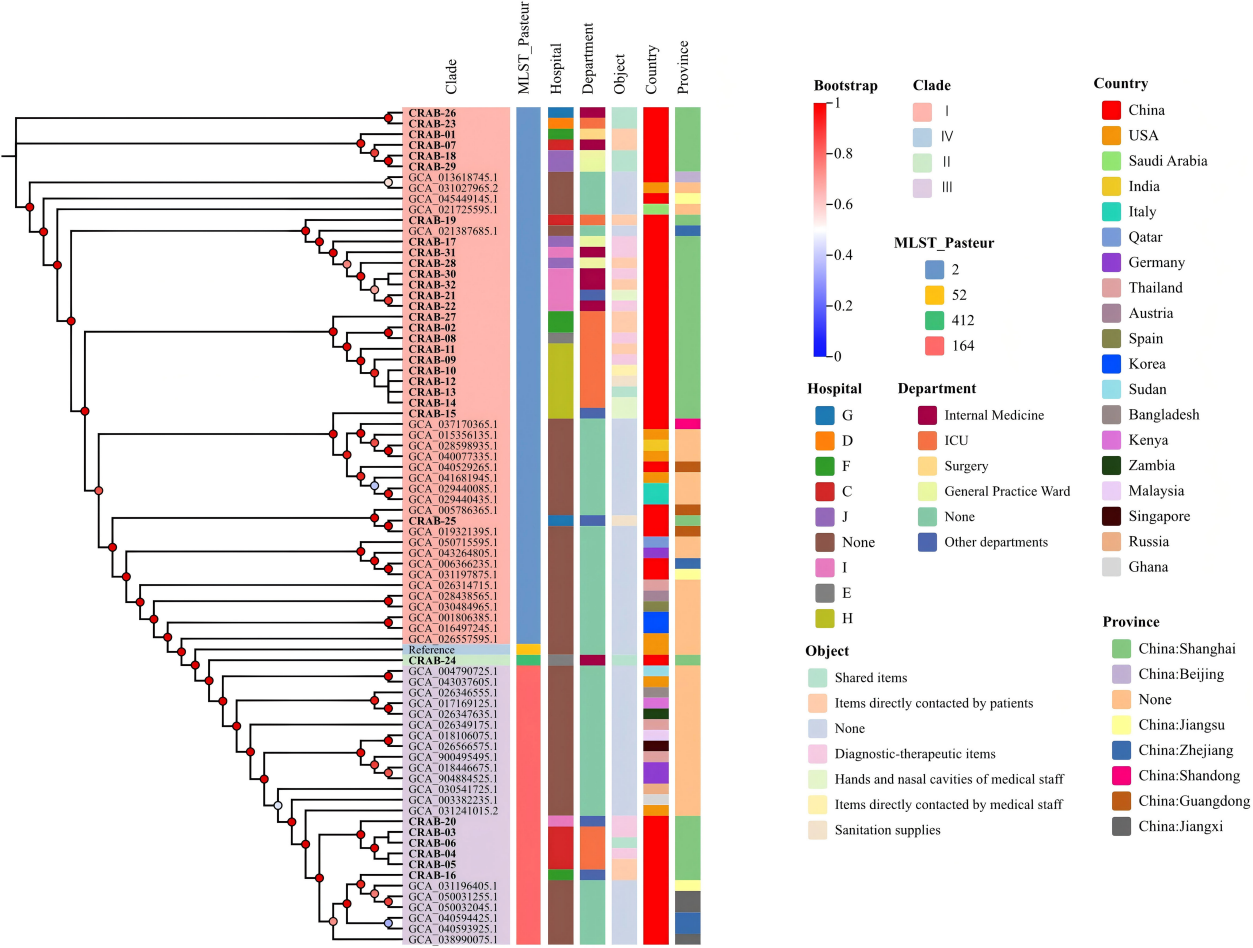
Phylogeny tree of *A. baumannii* isolates from 19 countries. Strains in bold represent the 32 CRAB isolates in this study, and the remaining strains were retrieved from public databases. The strain labeled “Reference” is the reference strain ATCC 19606. Circles indicate the bootstrap support values of each phylogenetic clade, with darker red corresponding to higher support values.

To characterize the genomic profiles of the 32 CRAB isolates collected in this study within local and global phylogenetic contexts, a total of 46 publicly available whole-genome sequences of *A. baumannii* recovered from 19 countries were incorporated for comparative analysis. Phylogenetic reconstruction of the combined dataset consisting of 78 *A. baumannii* genomes resolved four different clades (Clade IV designated as the reference group). Lineage I constituted the largest clade, all isolates of which belonged to ST2. This lineage encompassed strains isolated from 8 medical institutions, 5 departments, and 6 categories of object surfaces in the local region. Notably, isolate CRAB-21 recovered from healthcare workers in Hospital I exhibited close phylogenetic relatedness to CRAB-22 cultured from diagnostic-therapeutic items of the same hospital, with only a single SNP distinguishing their core genomes. A comparable pattern was observed in Hospital H: CRAB-14 derived from medical staff shared merely 0~6 core-genome SNPs with five environmental CRAB isolates (CRAB-09 to CRAB-13) collected from the same facility. Moreover, local strains within Clade I displayed tight genetic relatedness to isolates retrieved from the USA, as well as domestic strains from Guangdong and Zhejiang Provinces of China (**Figure 3**; **Supplementary Table 2**).

Clade III contained 26 *A. baumannii* isolates originating from 11 countries, all of which were assigned to ST164. Phylogenetic analysis revealed that the six ST164-type CRAB isolates recovered in the present study shared extremely close genetic relatedness. These strains also exhibited tight phylogenetic clustering with isolates from Jiangsu, Zhejiang and Jiangxi Provinces in China, with core-genome SNP differences ranging from 24 to 54. In contrast, they were genetically distant from strains isolated from the USA, Thailand and other foreign regions (**Figure 3**; **Supplementary Table 2**).

### 3.4 Antimicrobial resistance of *Acinetobacter baumannii*

**Figure 4:**
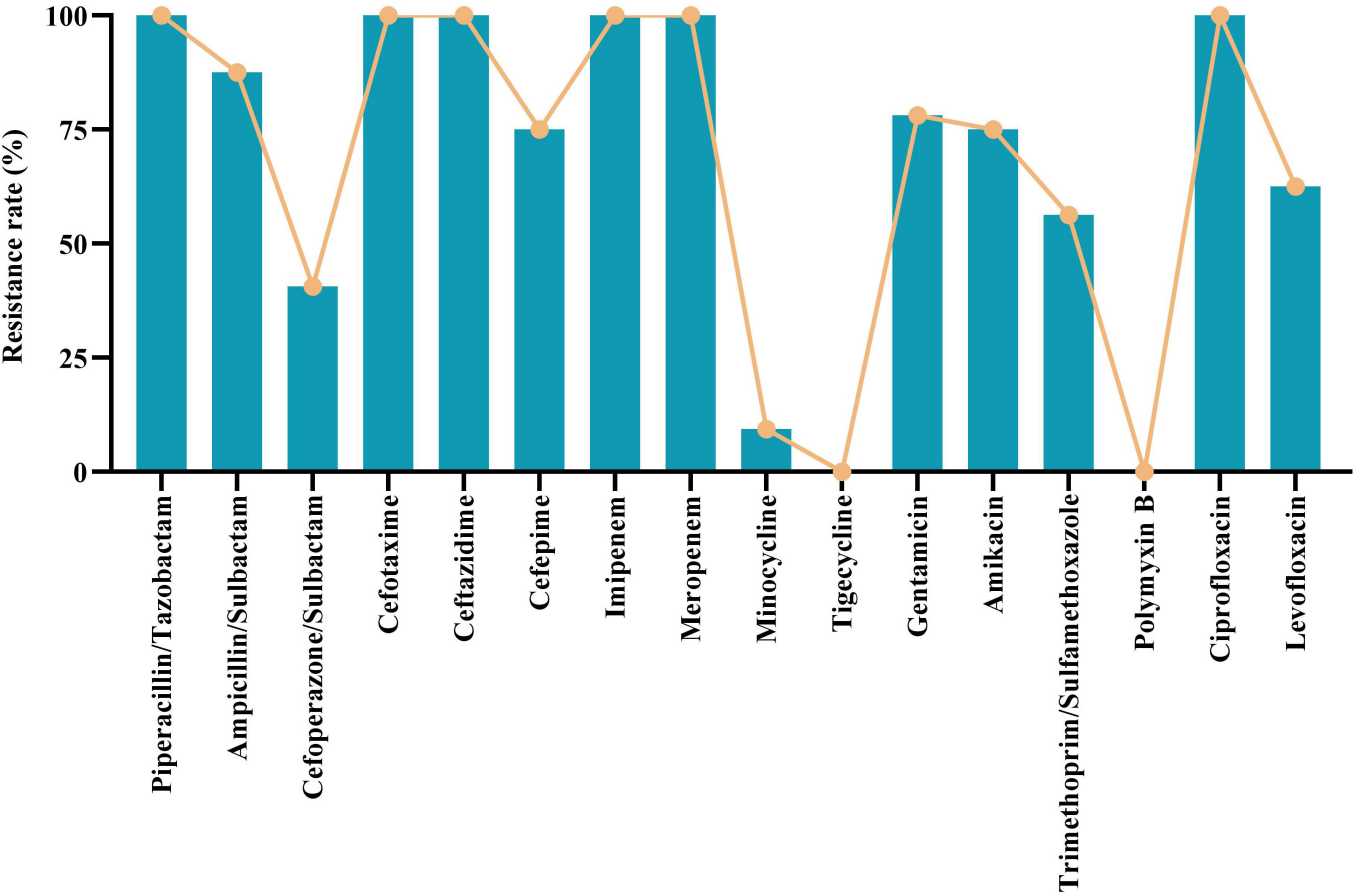
Antimicrobial resistance phenotypes of *A. baumannii* isolates. The bar chart shows the resistance rates of 32 CRAB strains against 16 antimicrobial agents.

The drug resistance results showed that in addition to being fully resistant to carbapenem antibiotics, the 32 CRAB strains were also 100% resistant to piperacillin/tazobactam, cefotaxime, ceftazidime, and ciprofloxacin. Meanwhile, CRAB strains also showed varying degrees of resistance to ampicillin/sulbactam, cefoperazone/sulbactam, cefepime, minocycline, gentamicin, amikacin, srimethoprim/sulfamethoxazole, and levofloxacin (**Figure 4**). However, CSAB strains were almost fully sensitive to the above antibiotics (only one isolate exhibited resistance to ampicillin/sulbactam). These results confirmed the significant difference in drug resistance profiles between CRAB and CSAB strains, and the potential hazard of CRAB’s multidrug resistance (**Table 2**). Importantly, no *A. baumannii* strains resistant to tigecycline or polymyxin B were found in this study.

**Table 2:** Antimicrobial resistance of CRAB and CSAB to various antimicrobial agents.

Antimicrobial family	Antimicrobial agents	Number of resistant isolates (%)		X^2^	P value
		CRAB (*n* = 32)	CSAB (*n* = 49)		
Penicillins	Piperacillin/tazobactam	32 (100.00)	0 (0.00)	81.000	<0.001*
	Ampicillin/sulbactam	28 (87.50)	1 (2.04)	61.510	<0.001*
Cephalosporins	Cefoperazone/sulbactam	13 (40.63)	0 (0.00)	23.712	<0.001*
	Cefotaxime	32 (100.00)	0 (0.00)	81.000	<0.001*
	Ceftazidime	32 (100.00)	0 (0.00)	81.000	<0.001*
	Cefepime	24 (75.00)	0 (0.00)	52.224	<0.001*
Carbapenems	Imipenem	32 (100.00)	0 (0.00)	81.000	<0.001*
	Meropenem	32 (100.00)	0 (0.00)	81.000	<0.001*
Tetracyclines	Minocycline	3 (9.38)	0 (0.00)	/	0.058
	Tigecycline	0 (0.00)	0 (0.00)	–	–
Aminoglycosides	Gentamicin	25 (78.13)	0 (0.00)	55.371	<0.001*
	Amikacin	24 (75.00)	0 (0.00)	52.224	<0.001*
Sulfonamides	Trimethoprim/ sulfamethoxazole	18 (56.25)	0 (0.00)	35.438	<0.001*
Polymyxins	Polymyxin B	0 (0.00)	0 (0.00)	–	–
Quinolones	Ciprofloxacin	32 (100.00)	0 (0.00)	81.000	<0.001*
	Levofloxacin	20 (62.50)	0 (0.00)	40.666	<0.001*

/, Fisher’s exact test (no X^2^ value); -, no comparison for identical resistance rates; *Bold value, *P* < 0.05.

**Figure 5:**
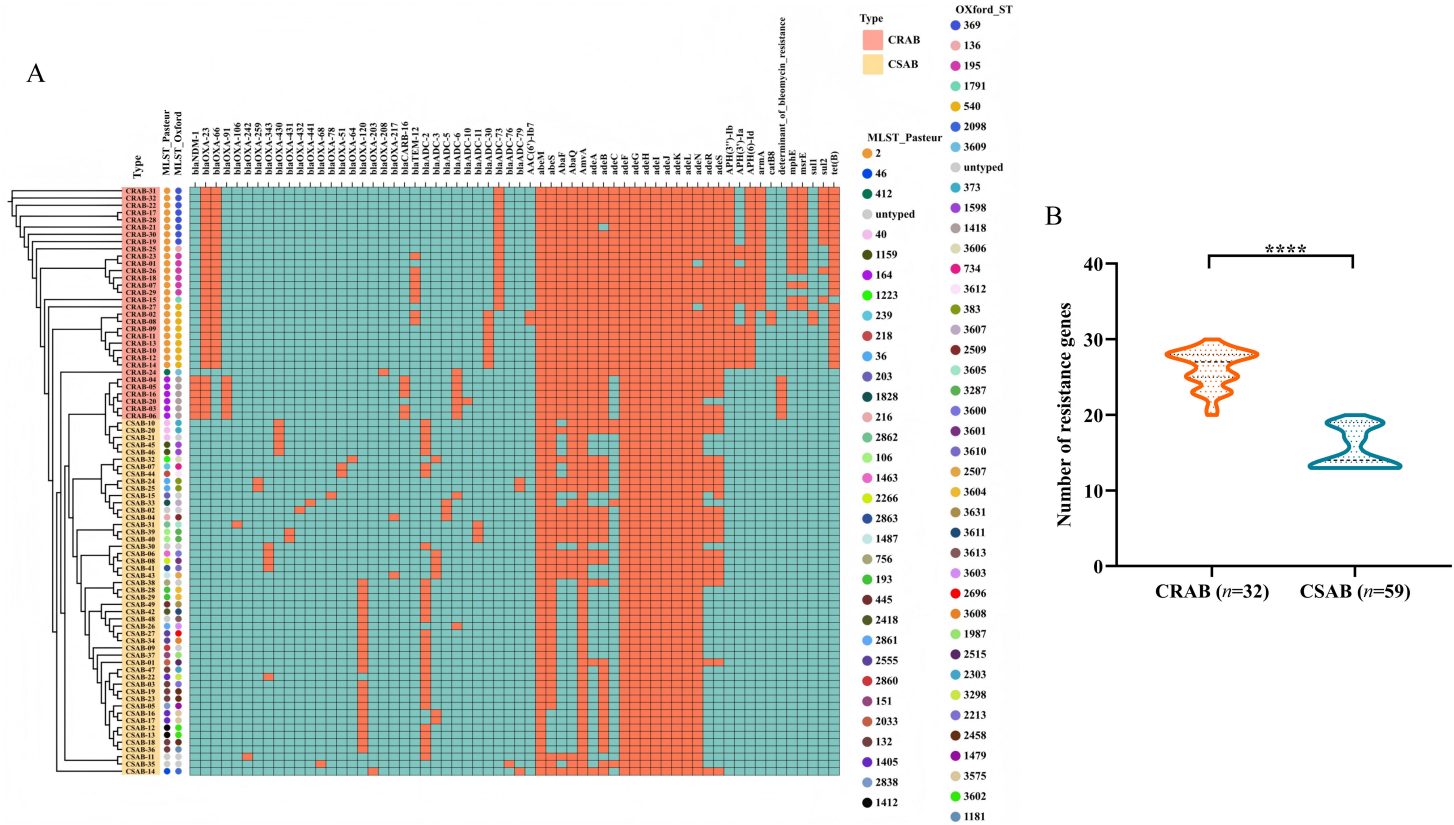
Distribution of resistance genes in *A. baumannii* isolates. **(A)** The clustered heatmap shows the carriage of resistance genes in CRAB and CSAB isolates. Orange squares indicate the presence of resistance genes, while green squares indicate their absence; **(B)** The violin plot compares the average number of resistance genes carried by CRAB and CSAB isolates. ****P < 0.0001.

To further elucidate the molecular mechanisms underlying antimicrobial resistance in *A. baumannii*, the carriage of resistance genes was characterized. As shown in **Figure 5A** and **Supplementary Table 3**, a total of 62 types of resistance genes were identified in the 81 *A. baumannii* isolates. The average number of resistance genes carried by CRAB isolates was significantly higher than that of CSAB (*P* < 0.05) (**Figure 5B**). Meanwhile, evident differences were observed in the composition of both acquired resistance genes and intrinsic determinants between the two groups. The carriage rates of the acquired carbapenem resistance genes *bla*_OXA-23_ reached 96.88% in CRAB isolates. Combined with MLST analysis, CRAB isolates belonging to ST2 simultaneously possessed *bla*_OXA-23_ and its corresponding lineage-specific intrinsic oxacillinase gene, *bla*_OXA-66_. Critically, six CRAB strains co-carrying *bla*_OXA-23,_ the acquired carbapenemases *bla*_NDM-1_ and the intrinsic *bla*_OXA-91_ gene all belonged to ST164. CRAB isolates also harbored a variety of acquired resistance genes related to aminoglycoside, tetracycline, macrolide, and sulfonamide antibiotics, with carriage rates of *APH(3’’)-Ib* (78.13%), *APH(6)-Id* (78.13%), *armA* (53.13%), *APH(3’)-Ia* (40.63%), *mphE* (46.88%), *msrE* (46.88%), *tet(B)* (75%), and *sul2* (31.25%) respectively. Instead, CSAB isolates lacked all the aforementioned genes. In addition to the universally shared efflux pump genes (*abeM*, *amvA*, and *adeF*), CSAB isolates specifically presented with the intrinsic resistance determinants *bla*_OXA-120_ and *bla*_ADC-2_.

A concordance analysis between resistance phenotypes and genotypes was performed for the 81 *A. baumannii* isolates. The drug classes included in this analysis were β-lactams, tetracyclines, aminoglycosides, and sulfonamides. The kappa coefficients for *bla*_OXA-23_ with piperacillin/tazobactam, ampicillin/sulbactam, cefotaxime, ceftazidime, cefepime, imipenem, and meropenem were all greater than 0.8, demonstrating near-perfect agreement. *APH(3’)-Ia* and *armA* with aminoglycosides, and *sul2* with trimethoprim/sulfamethoxazole showed substantial agreement, with kappa coefficients ranging between 0.6 and 0.8. In contrast, poor concordance was observed between *tet(B)* and minocycline, yielding a kappa coefficient below 0.2 (**Supplementary Table 4**).

### 3.5 Plasmid prediction of *Acinetobacter baumannii*

**Figure 6:**
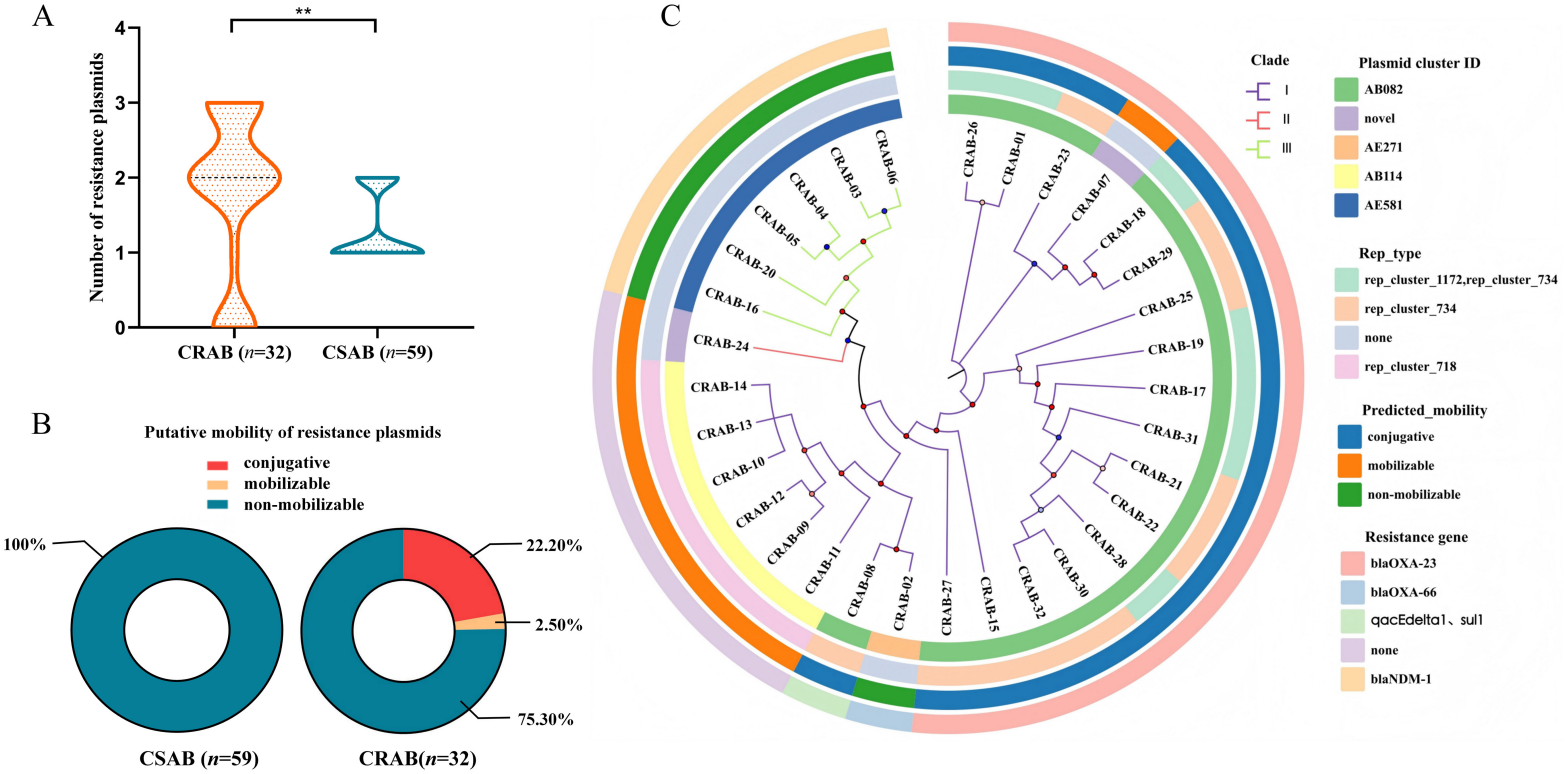
Plasmid prediction in *A. baumannii* isolates. **(A)** The violin plot compares the average number of predicted plasmids carrying resistance genes between CRAB and CSAB, **P < 0.01; **(B)** The two donut charts separately show the proportions of resistance gene-carrying plasmids with distinct mobility features in CSAB (left) and CRAB (right) isolates based on bioinformatic analyses; **(C)** The phylogenetic tree illustrates some of predicted plasmids and matching antimicrobial resistance gene profiles identified in 32 CRAB isolates. The annotations are (from inner to outer ring): Plasmid cluster ID, Rep_type, Predicted_mobility and Resistance gene of CRAB isolates. Clades I–III are distinguished by branches in distinct colors. Circles indicate the bootstrap support values of each phylogenetic clade, with darker red corresponding to higher support values.

The study further characterized the carriage of key plasmids across all *A. baumannii* isolates via bioinformatic analysis. In silico prediction showed that the number of plasmids carrying resistance genes in CRAB was significantly higher than that in CSAB (*P* < 0.05) (**Figure 6A**), with marked differences in plasmid profiles between the two groups. Among the plasmids in CRAB, 75.30% were putatively non-mobile plasmids, 22.20% were putatively conjugative plasmids, and 2.50% were classified as mobilizable plasmids, while all CSAB plasmids were predicted non-mobilizable (**Figure 6B**). The carriage of resistance genes in CRAB plasmids was inferred via bioinformatic tools. Prediction results indicated that *bla*_OXA-23_ gene was likely located on conjugative or mobilizable plasmids in 68.00% (17/25) of strains within Clade I. In contrast, *bla*_NDM-1_ gene carried by all ST164-type strains may reside on non-mobile plasmids. Notably, the study also found that the plasmid of CRAB-08 was predicted to simultaneously carried the sulfonamide resistance gene *sul1* and the quaternary ammonium compound disinfectant resistance gene *qacEdelta1*, both of which were putatively located on conjugative plasmids (**Figure 6C**).

### 3.6 Virulence genes in *Acinetobacter baumannii*

**Figure 7:**
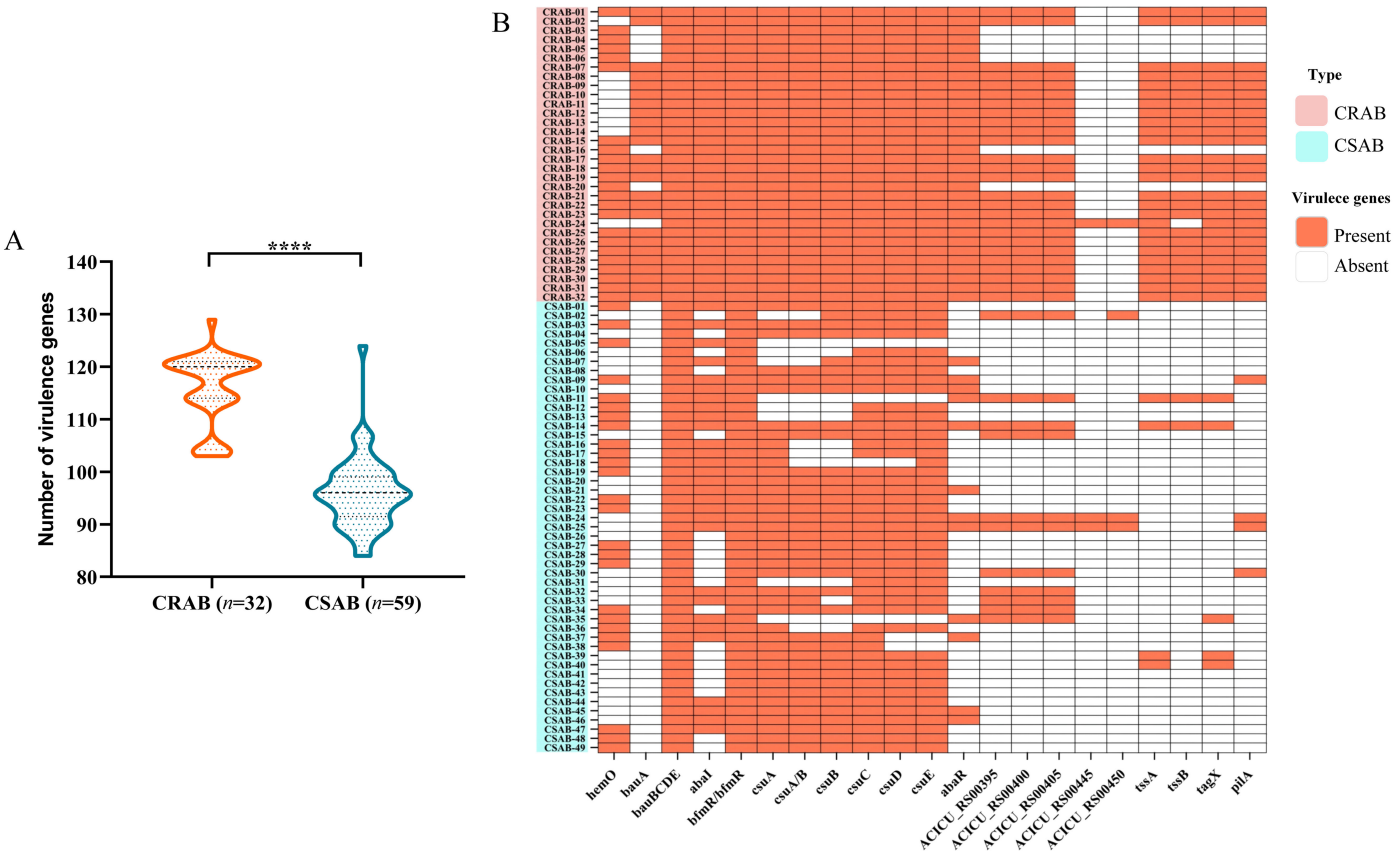
Distribution of virulence genes in *A. baumannii* isolates. **(A)** The violin plot compares the average number of virulence genes carried by CRAB and CSAB isolates. ****P < 0.0001 **(B)** The clustered heatmap shows the carriage of virulence genes in CRAB and CSAB isolates. Orange squares indicate the presence of virulence genes, while white squares indicate their absence.

The virulence genes of CRAB and CSAB that exert virulence through different mechanisms were characterized. The results showed that the average number of virulence genes carried by CRAB was significantly greater than that by CSAB, with an average of 116 and 96, respectively (**Figure 7A**). Comparing virulence genes involved in various biological functions, the carriage rates of genes related to iron uptake (*bauA*), biofilm formation (csu operon, *abaI/R* system), secretion system (*tssA/B*), and adhesion (*pilA*) in CRAB were all significantly higher than those in CSAB (*P* < 0.05) (**Table 3** and **Figure 7**).

**Table 3:** Virulence gene carriage and functional classification of CRAB and CSAB.

Function	Virulence genes	Number of strains carrying virulence genes (%)		X^2^	P value
		CRAB (*n* = 32)	CSAB (*n* = 49)		
Iron uptake	hemO	23 (71.88)	25 (51.02)	3.487	0.062
	bauA	25 (78.13)	0 (0.00)	55.371	<0.001*
	bauB, bauC, bauD, bauE	32 (100.00)	49 (100.00)	–	–
Biofilm formation	abaI	32 (100.00)	29 (59.18)	17.344	<0.001*
	bfmR, bfmS	32 (100.00)	49 (100.00)	–	–
	csuA	32 (100.00)	40 (81.63)	/	0.010*
	csuA/B	32 (100.00)	36 (73.47)	10.113	0.001*
	csuB	32 (100.00)	37 (75.51)	/	0.002*
	csuC	32 (100.00)	45 (91.84)	/	0.149
	csuD	32 (100.00)	43 (87.76)	/	0.076
	csuE	32 (100.00)	44 (89.80)	/	0.151
	abaR	32 (100.00)	12 (24.49)	44.482	<0.001*
Immune evasion	ACICU_RS00395	26 (81.25)	11 (22.45)	26.974	<0.001*
	ACICU_RS00400	26 (81.25)	11 (22.45)	26.974	<0.001*
	ACICU_RS00405	26 (81.25)	11 (22.45)	26.974	<0.001*
	ACICU_RS00445	1 (3.13)	2 (4.08)	/	1.000
	ACICU_RS00450	1 (3.13)	3 (6.12)	/	1.000
Secretion System	tssA	26 (81.25)	4 (8.16)	44.342	<0.001*
	tssB	25 (78.13)	2 (4.08)	47.758	<0.001*
	tagX	25 (78.13)	5 (10.20)	38.295	<0.001*
Adherence	pilA	26 (81.25)	4 (8.16)	44.342	<0.001*

/, Fisher’s exact test (no X^2^ value); -, no comparison for identical carriage rates; *Bold value, *P* < 0.05.

## 4 Discussion

Since the discovery of *A. baumannii*, its detection rate in medical institutions has been increasing. Coupled with its significant resistance to multiple antibiotics, this bacterium has gradually evolved into an important pathogen threatening public health security (Sharma and LakhanpalAcinetobacter, 2025). As a key representative of carbapenem-resistant bacteria, CRAB strains poses an urgent global public health challenge, with its transmission being particularly prominent in medical institutions (Liu H. et al., 2024; Karakonstantis et al., 2025). CRAB strains exhibit high prevalence in nosocomial infections, attributed to its strong environmental adaptability—it can colonize various medical supplies and survive on dry surfaces for months, and a prior CRAB colonization history increases the subsequent infection risk by 8-fold (Medioli et al., 2022). Of note, CRAB strains infection leads to more severe clinical outcomes compared with CSAB strains. Thus, clarifying the distribution, drug resistance characteristics, and transmission patterns of CRAB and CSAB isolates in medical institutions is essential to guide the prevention and control of *A. baumannii*, especially CRAB isolates. This study systematically analyzed the distribution, ST molecular typing, drug resistance profile, plasmid carriage, and virulence gene characteristics of CRAB and CSAB strains in 10 medical institutions in Pudong New Area, providing targeted scientific evidence for healthcare-associated infection control in this region.

This study demonstrated significant differences in the distribution of CRAB and CSAB strains across medical institutions, departments, and sampling objects. CRAB isolates were highly concentrated in ICU, predominantly isolated from patient-direct contact items and diagnostic-therapeutic equipment. In contrast, CSAB isolates were evenly distributed in other departments, with shared items and medical staff’s hands/nasal cavities serving as the main reservoirs. These findings confirm that the ICU is a high-risk area for CRAB isolates colonization, and patient-direct contact items are likely core vehicles for the spread of environmental CRAB (Ng et al., 2018; Doughty et al., 2023; Liu L. et al., 2024; Wang B. et al., 2025). As a specialized unit for critically ill patients, ICUs feature immunocompromised individuals, frequent invasive procedures, and high-intensity antimicrobial use—collectively fostering a microenvironment conducive to CRAB strains colonization and persistence. Furthermore, the detection rates of CRAB isolates varied significantly among different hospitals, which may be closely related to disinfection methods, implementation intensity of infection control measures, and management level of antimicrobial use in each hospital. Additionally, *A. baumannii* detection rates were relatively high on surfaces of faucets, pillows, and bedding-items with high-frequency contact and easy neglect in cleaning/disinfection—further verifying that hospital environmental surfaces serve as key *A. baumannii* reservoirs. Based on these differences, targeted prevention and control strategies were warranted: (1) Conduct intensive disinfection for ICUs and patient-contact items; (2) Optimize infection control procedures in hospitals with high CRAB detection rates; (3) Improve hand hygiene training for medical staff to reduce the circulation of CSAB and remain alert to potential dissemination of CRAB between humans and environmental surfaces.

ST2 CRAB strains have been well-documented as the predominant clonal lineage driving global CRAB infection outbreaks, largely due to its robust environmental adaptability and enhanced resistance gene-carrying capacity (Nawfal Dagher et al., 2019; Yu et al., 2021; Brek et al., 2025; Zhang et al., 2025). In this study, CRAB isolates were restricted to 3 STs, with ST2 accounting for 78.13%, which is consistent with the globally dominant CRAB epidemic clone (Valcek et al., 2022; Mat Ghani et al., 2025). Phylogenetic analysis revealed that ST2 strains from 8 hospitals and 5 departments belonged to the same evolutionary clade. This high genetic relatedness suggests the possibility of inter-hospital and intra-hospital clonal dissemination of CRAB strains, whereas definitive transmission chains need further analysis combined with epidemiological data. It is noteworthy that CRAB isolates detected from medical staff’s hands/nasal cavities shared close genetic proximity with strains from items directly contacted by patients and diagnostic-therapeutic items. This evolutionary characteristic implies the potential transmission routes of CRAB between healthcare personnel and the medical environment, underscoring the previous conclusion that “hand hygiene is a crucial measure to interrupt the potential nosocomial transmission of *A. baumannii*.” *(*Garnacho-Montero et al., 2015; Weinberg et al., 2020). In addition, the phylogenetic tree integrated with public database genomes displayed that the local ST2 strains showed low core-genome SNP distances to domestic isolates from high-prevalence provinces (Guangdong and Zhejiang) and tight genomic clustering with the USA isolates, which may imply the potential transregional mobility of ST2. Intriguingly, although the ST164-type CRAB isolates co-harboring two carbapenemase genes in this study exhibited substantial genetic differentiation from overseas strains, they shared high genetic relatedness with isolates from neighboring provinces. This reminds us that routine surveillance should not only guard against the persistent threat of international clones like ST2, but also continuously monitor ST164 clones with significant resistance potential to prevent their dissemination.

Antimicrobial susceptibility testing showed typical multidrug resistance in CRAB strains, whereas CSAB strains were sensitive to most tested antibiotics. This disparity appeared to be closely associated with the distinct resistance gene patterns. In this study, the carriage rate of the acquired carbapenemase *bla*_OXA-23_ in CRAB was as high as 96.88%, which is the core gene mediating *A. baumannii* resistance to carbapenem antibiotics (Sharma et al., 2022; Ababneh et al., 2025; Mat Ghani et al., 2025). The β-lactamase encoded by *bla*_OXA-23_ can effectively hydrolyze carbapenem antibiotics, which is the main molecular mechanism for the prevalence of CRAB strains (Liu et al., 2022). More alarmingly, as mentioned above, six ST164 CRAB strains simultaneously harboring *bla*_OXA-23_, *bla*_NDM-1_ and the chromosome-encoded intrinsic oxacillinase *bla*_OXA-91_.While *bla*_OXA-91_ represents an intrinsic background marker for ST164, the horizontal co-acquisition of the exogenous class D (*bla*_OXA-23_) and class B (*bla*_NDM-1_) carbapenemases amplifies the resistance potential. Liu et al. reported that ST164 isolates had higher MIC50/MIC90 values for carbapenem antibiotics than global clone 2. A comparison of genomes from 26 countries revealed that ST164 has independently evolved carbapenem resistance multiple times (Liu H. et al., 2024). In addition, a study by Wang et al. demonstrated that ST164 CRAB isolates exhibited a significantly stronger biofilm-forming capacity *in vitro* than ST2 isolates (Wang et al., 2026). Existing research also revealed that ST164 strains produce pyomelanin to achieve substantially elevated survival under UV irradiation (Zhao et al., 2022). Collectively, these studies provide stronger evidence that CRAB strains belonging to ST164 represent a clone with potential risks, warranting global attention and focused surveillance (Liu H. et al., 2024; Wang K. et al., 2025). No *A. baumannii* strains resistant to tigecycline or polymyxin B were found, and this near-zero resistance conclusion is consistent with the research results of Hou, Hu, and others (Hu et al., 2024; Chen et al., 2025; Hou et al., 2025). Tigecycline and polymyxin B remain important clinical drugs for the treatment of CRAB strains infections, providing certain guarantees for the selection of clinical treatment regimens. Nevertheless, vigilance is required to monitor the emergence of resistance driven by the long-term clinical use of these agents.

As pivotal carriers mediating horizontal transfer of resistance genes, plasmids directly affect the transmission risk of drug-resistant bacteria (Acman et al., 2022; Kilbas et al., 2023). Theoretically, conjugative plasmids achieve horizontal transfer through direct bacteria-to-bacteria contact, serving as a key vehicle for the rapid dissemination of resistance genes. This notion aligns with the view proposed by Ying et al. that. “horizontal transmission of the *bla*_OXA-23_ gene is an important driver of CRAB nosocomial epidemics” (Liu et al., 2015; Ying et al., 2025). Our bioinformatic analyses also suggested that nearly 70.00% of CRAB strains harbored the *bla*_OXA-23_ gene on predicted conjugative or mobilizable plasmids, which raised the possibility of horizontal spread of this gene via mobile genetic elements. Furthermore, a unique feature was identified in strain CRAB-08: its putatively conjugative plasmid likely co-carries the sulfonamide resistance gene *sul1* and the quaternary ammonium compound disinfectant resistance gene *qacEdelta1.* This genomic feature predicts the potential for both “therapeutic resistance” and “environmental tolerance” capabilities in this strain. Quaternary ammonium compound disinfectants were widely used in hospital environmental disinfection. Carriage of the *qacEdelta1* gene may potentially reduce the bactericidal efficacy of disinfectants against *A. baumannii*, thereby enhancing its colonization and transmission probability within healthcare settings. Naturally, further phenotypic assays targeting disinfectant resistance are required to verify this speculation. Nevertheless, this finding still reminds us that long-term exclusive use of a certain type of disinfectant may induce selective pressure on drug-resistant strains, highlighting the need to optimize hospital disinfection protocols to avoid the development of disinfectant resistance.

This study has several limitations that should be acknowledged. First, the sample collection period was relatively short, resulting in a relatively small number of strains. Second, no clinical infection case data were integrated, making it impossible to clarify the direct association between the characteristics of environmental CRAB and CSAB and clinical infections. Third, epidemiological information regarding patient movement and layout was unavailable, preventing further verification of the speculated transmission chains. Fourth, no assays including gene expression quantification, biofilm formation detection, animal infection models or other virulence phenotypic validations were performed in this study, so present results only demonstrated differences in the carriage abundance of virulence-associated genes among isolates.

## 5 Conclusions

In summary, this study systematically clarified the significant differences between CRAB and CSAB strains in healthcare institution environments across multiple dimensions. ICU environments and items directly contacted by patients were identified as key high-risk links for CRAB prevention and control. ST2 and ST164 were the clones of CRAB isolates that deserve the greatest attention. CRAB strains exhibited stronger multidrug-resistant phenotypes compared to CSAB strains. Bioinformatic evidence suggested the co-occurrence of antimicrobial-resistance and disinfectant-resistance-associated genes in some CRAB isolates, which may increase their persistence and dissemination potential. In conclusion, it’s critical for blocking the potential transmission routes of drug-resistant *A. baumannii* to strengthen the disinfection and management of high-frequency contact surfaces in hospitals and conduct precise monitoring of dominant clones.

## Data Availability

The datasets presented in this study can be found in online repositories. The names of the repository/repositories and accession number(s) can be found below: https://www.ncbi.nlm.nih.gov/, PRJNA1390075.
